# Prevalence of anaemia and associated risk factors among pregnant women attending antenatal care in Gulu and Hoima Regional Hospitals in Uganda: A cross sectional study

**DOI:** 10.1186/s12884-016-0865-4

**Published:** 2016-04-11

**Authors:** Gerald Obai, Pancras Odongo, Ronald Wanyama

**Affiliations:** Department of Physiology, Faculty of Medicine, Gulu University, P.O Box 166, Gulu, Uganda; Department of Internal Medicine, Hoima Regional Hospital, P.O Box 5, Hoima, Uganda; Department of Biochemistry, Faculty of Medicine, Gulu University, P.O Box 166, Gulu, Uganda

**Keywords:** Anaemia, Gulu, Hoima, Hospital, Pregnant women, Prevalence

## Abstract

**Background:**

Anaemia is a public health problem affecting over 1.62 billion people globally. It affects all age groups of people and is particularly more prevalent in pregnant women. Africa carries a high burden of anaemia; in Uganda 24 % of women of child bearing age have anaemia. Pregnant women living in poverty are at greater risk of developing iron deficiency anaemia. The objective of this study was to determine the prevalence of anaemia and the associated risk factors in pregnant women attending antenatal care at Gulu and Hoima Regional Hospitals in Northern and Western Uganda respectively.

**Methods:**

We conducted a cross sectional study in Gulu and Hoima Regional Hospitals from July to October 2012. Our study participants were pregnant women attending antenatal care. Socio-demographic data were collected using structured questionnaires and blood samples were collected for haemoglobin estimation. Haemoglobin concentration was determined using an automated analyzer closed mode of blood sampling. Data were analysed using Stata version 12. Odds ratio was used as a measure of association, with 95 % confidence interval; and independent risk factors for anaemia were investigated using logistic regression analyses. Ethical approval was obtained from Gulu University Research Ethics Committee and written informed consent was obtained from each study participant.

**Results:**

The overall prevalence of anaemia was 22.1 %; higher in Gulu (32.9 %) than in Hoima (12.1 %), *p* < 0.001. In Gulu, the prevalence of mild anaemia was 23 %, moderate anaemia was 9 %, and severe anaemia was 0.8 %, while in Hoima, the prevalence of mild anaemia was 9 %, moderate anaemia was 2.5 %, and severe anaemia was 0.5 %. Independent risk factors for anaemia were: being a housewife [Adjusted Odds Ratio (AOR) = 1.7, 95 % CI: 1.05–2.68]; and being a resident in Gulu (AOR = 3.6, 95 % CI: 2.41–5.58).

**Conclusion:**

The prevalence of anaemia in pregnant women in Gulu is higher than in Hoima. Amongst pregnancy women, being a housewife is an independent risk factor for anaemia. Greater efforts are required to encourage early antenatal attendance from women in these at risk groups. This would allow iron and folic acid supplementation during pregnancy, which would potentially reduce the prevalence of anaemia.

**Electronic supplementary material:**

The online version of this article (doi:10.1186/s12884-016-0865-4) contains supplementary material, which is available to authorized users.

## Background

Anaemia is a global public health problem affecting over 1.62 billion people [1]. It affects all age groups of people but pregnant women and children are more vulnerable. Iron deficiency is the leading cause of anaemia among pregnant women globally [2]. Other causes of anaemia in pregnancy are heavy blood loss as may occur during menstruation and parasitic infections, conditions such as malaria and HIV which lower blood haemoglobin (Hb) concentrations, and micronutrient deficiencies [1]. Low intake and poor absorption of iron especially during growth and pregnancy when iron requirements are higher remain risk factors for anaemia [3]. The World Health Organisation defines anaemia in pregnant women as Hb concentration less than 11.0 g/dl [4]. In pregnant women, anaemia increases risk for maternal and child mortality and has negative consequences on the cognitive and physical development of children [5], and on work productivity [6, 7]. Severe anaemia is associated with fatigue, weakness, breathlessness, dizziness, drowsiness and perceived paleness of the skin [8]. In the developing world, anaemia is a priority nutritional problem because of the economic, social, and other negative consequences associated with it [9]. Africa carries a high burden of anaemia with a prevalence of 65.8 % among pregnant women [1]. In Uganda, the prevalence of anaemia among women of child bearing age has been reported to be 24 % overall, and 13.1 and 18.8 % in Northern and Western Uganda, respectively [10].

Poverty is one of the risk factors for iron deficiency in pregnant women [11, 12], and given the fact that Northern region is the poorest region in Uganda [13] with high rates of malnutrition, the problem of anaemia cannot be underestimated. Despite the known consequences of anaemia in pregnancy, there is scanty information on the prevalence of anaemia in pregnant women in Northern Uganda since the end of the Lord’s Resistance Army (LRA) rebellion in 2006. The two decades of civil war in Northern Uganda led to the destruction of social services and left the majority of the population in poverty. Western Uganda on the other hand remained peaceful during the same period. It was therefore necessary to compare the prevalence of anaemia in the two regional hospitals. This would give an insight into the long term effect of the war on women of child bearing age. The objective of this study was to determine the prevalence of anaemia and associated risk factors among pregnant women attending antenatal care at Gulu and Hoima Regional Hospitals so that evidence-based interventions can be put in place.

## Methods

### Study design and setting

We conducted a cross sectional study at Gulu and Hoima Regional Hospitals from July to October 2012. Gulu and Hoima Regional Hospitals are located in Northern and Western Uganda, respectively. Gulu district headquarters are located approximately 340 km North of Uganda’s capital city, Kampala. The population of Gulu District is 443,733 [14]. The coordinates of the district are: 02 45 N, 32 00E. Hoima district headquarters are located approximately 230 km Northwest of Kampala. The population of Hoima district is 573,903 [14]. The coordinates of the district are: 01 24 N, 31 18E.

### Study population

Our study participants were pregnant women attending antenatal care in the two regional hospitals. All pregnant women who consented to the study and who reported that they did not have sickle cell disease were eligible to participate in the study. This is because persons who have sickle cell disease usually have lower Hb levels than the normal persons.

### Sample size and sampling procedures

The sample size for this study was calculated using Kish Leslie formula. We considered 95 % confidence interval, 5 % margin of error, and 45 and 64 % prevalence of anaemia for Gulu (Northern Uganda) and Hoima (Western Uganda), respectively as was reported by Ugandan Demographic and Health surveys [15]. We factored a 10 % non response rate in the sample size calculation. The two hospitals were purposively sampled since they are regional referral hospitals in the study areas with high patient load, from different backgrounds and settings. We used simple random sampling to select the study participants. Each day, before the provision of health education, the names of every pregnant woman attending the antenatal clinic was taken. Fifty percent of the women were then randomly selected until the required sample size was attained. The probability sampling was employed to avoid selection bias.

### Socio-demographic data

Data on socio-demographic characteristics were collected using structured questionnaires (Additional file 1). The questionnaire was pre-tested in an area with similar settings to those of the study hospitals. The questionnaire was then refined to further improve its validity and reliability. The questionnaires were both in English and the local languages. Trained research assistants who were fluent in both English and the local language (Luo or Lunyoro) conducted face-to-face interviews with the pregnant women. The interviews were conducted in privacy to maintain confidentiality, within the hospital premises.

### Collection and analysis of blood samples

The vein puncture site was cleaned using a swab containing 70 % alcohol and using aseptic methods, an appropriate vein was identified and a hypodermic needle introduced into the vein. About 3–4 ml of venous blood was drawn into a syringe and then transferred into a sterile vacutainer containing EDTA and transported to the laboratory for analysis. Trained laboratory technicians did the analyses both in Gulu and Hoima Regional Hospitals. Laboratory analysis was done using an automated analyser, (Celltac, Automated Haematology Analyzer, MEK-6400. NIHON KOHDEN). The manufacturer supplied controls were run every morning to ensure that the analyser was operating within 2.0 standard deviations. The closed mode of blood sampling was used; the analyser automatically sampled blood, processed, analysed and printed out the haemoglobin concentration levels. Pregnant women with haemoglobin concentration of less than 11.0 g/dl were categorised as anaemic. Anaemia was considered severe when haemoglobin concentration was less than 7.0 g/dL, moderate when haemoglobin was between 7.0 and 9.9 g/dL, and mild from 10 to 10.9 g/dL [4].

### Statistical analysis

Both the laboratory and questionnaire data were checked and cleaned for completeness and consistency. Participants with missing data on haemoglobin level were excluded from the analyses. Statistical analysis was performed using Stata version 12. Quantitative variables were categorised into groups basing on either biologically recognised groupings like trimester in pregnancy or societal recognised groupings like education levels. Descriptive statistics was employed for the analysis of demographic data. We used odds ratios as a measure of association, with a 95 % confidence interval. Variables with p-values <0.2 at bivariable analysis and those with biological plausibility with respect to anaemia were put into backward stepwise multivariable logistic regressions to determine the independent predictors for anaemia in pregnancy. Statistical significance was set at *P* < 0.05.

### Ethical considerations

The study was approved by Gulu University Research Ethics Committee. Written informed consent was obtained from each study participant before data collection. Privacy and confidentiality was maintained throughout the study process.

## Results

A total of 743 pregnant women took part in this study for a response rate of 91.1 %. Some participants withdrew from the study at the point of blood collection. A large majority of the study participants were below the age of 25 years (Table 1). A total of 164 (22.1 %) women were anaemic. The prevalence of anaemia in Gulu was 32.9 % (117/356) and that in Hoima was 12.1 % (47/387). The prevalence of mild, moderate and severe anaemia in Gulu were 23, 9, and 0.8 % respectively; and in Hoima were 9, 2.5, and 0.5 %, respectively (Table 2). In the bivariable analysis, anaemia was significantly associated with the level of education attained, occupation, being in or out of school, and being a resident of Gulu district (Table 3). Being a housewife (AOR = 1.7, 95 % CI: 1.05–2.68) and a resident in Gulu districts (AOR = 3.6, 95 % CI: 2.41–5.58) were independent risk factors for anaemia (Table 4).

**Table 1:** Socio-demographic characteristics of the study participants (*N* = 743)

	Hoima (*N* = 387)		Gulu (*N* = 356)	
Characteristic	Number	Percent	Number	Percent
Age				
≤19	102	26.4	97	27.3
20–24	116	30.0	127	35.7
25–29	91	23.5	87	24.4
30–34	54	14.0	32	9.0
35–39	22	5.7	9	2.5
>39	2	0.5	4	1.2
Education level				
No education	26	6.7	23	6.5
Primary	186	48.1	204	57.3
Secondary	149	38.5	104	29.2
Tertiary	26	6.7	25	7.0
Still in school				
Yes	26	6.7	13	3.7
No	361	93.3	343	96.3
Marital status				
Married	321	83.0	332	93.3
Single	60	15.5	19	5.3
Widowed	1	0.3	3	0.8
Separated/divorced	5	1.3	2	0.6
Occupation				
Farming	130	33.6	92	25.8
Trader	92	23.8	62	17.4
Formal employment	51	13.2	32	9.0
Handicraft	0	0.0	10	2.8
Housewife	114	29.4	160	44.9
Household size				
1–5	286	73.9	249	69.9
6–10	94	24.3	96	27.0
>10	7	1.8	11	3.1
Wealth index				
Lowest	278	71.8	184	51.7
Second lowest	85	22.0	149	41.9
Medium	15	3.9	17	4.8
High	9	2.3	6	1.7
Residence				
Rural	164	42.4	137	38.5
Urban	223	57.6	219	61.5
Trimester				
First	42	10.8	13	3.7
Second	200	51.7	105	29.5
Third	145	37.5	238	66.8
Gravidity				
1–4	309	79.8	301	84.6
>4	78	20.2	55	15.4
Delivery gap				
Never delivered	130	33.6	120	33.7
1–11 months	10	2.6	12	3.4
12–24	75	19.4	41	11.5
25–36	60	15.5	79	22.2
>36	112	28.9	104	29.2

**Table 2:** Anaemia prevalence in Gulu and Hoima districts

District	Number	Percent
Gulu		
Total anaemia	117	32.9
Mild anaemia	82	23.0
Moderate anaemia	32	9.0
Severe anaemia	3	0.8
Hoima		
Total anaemia	47	12.1
Mild anaemia	35	9.0
Moderate anaemia	10	2.5
Severe anaemia	2	0.5

**Table 3:** Chi- square tests of anaemia and associated risk factors among pregnant women in Gulu and Hoima Regional Hospitals

Variable	Anaemic		Non anaemic		P-value
	Number	Percentage	Number	Percentage	
Age					
≤19	47	23.6	152	76.4	
20–24	48	19.8	195	80.2	0.62
25–29	42	23.6	136	79.4	
30–34	21	24.4	65	75.6	
35–39	4	12.9	27	87.1	0.58^b^
>39	2	33.3	4	66.7	
Education					
No education	11	45.8	13	54.2	
Primary	87	22.8	295	77.2	0.01^a^
Secondary	59	20.9	224	79.1	
Higher	7	13.0	47	87.0	
Still in school					
Yes	3	7.7	36	92.3	0.03^b^
No	161	22.9	543	77.1	0.03^a^
Occupation					
Farmer	38	17.1	184	81.9	
Trader	31	20.1	123	79.9	0.03^a^
Formal employment	15	18.1	68	81.9	
Handicraft	3	30.0	7	70.0	0.03^b^
Housewife	77	28.1	197	71.9	
Residence					
Rural	67	22.3	234	77.7	0.9
Urban	97	22.0	345	78.0	
Marital status					
Single	14	17.7	65	82.3	0.2
Married	145	22.2	508	77.8	
Widowed	2	50.0	2	50.0	0.1^b^
Separated/divorced	3	42.9	4	57.1	
District					
Hoima	47	12.1	340	87.9	<0.01^a^
Gulu	117	32.9	239	67.1	
Trimester					
First	8	14.6	47	85.4	0.2
Second	63	20.7	242	79.3	
Third	93	24.3	290	75.7	
Gravidity					
1–4	133	21.8	477	78.2	0.7
>5	31	23.3	102	76.7	
Delivery gap (months)					
Never delivered	61	24.4	189	75.6	0.8
1–11	5	22.7	17	77.3	
12–24	23	19.8	93	80.2	
25–36	31	22.3	108	77.7	
>36	44	20.4	172	79.6	
Household size					
1–5	111	20.8	424	79.2	0.3
6–10	48	25.3	142	74.7	
>10	5	27.8	13	72.2	
Wealth index					
Lowest	100	21.7	362	78.3	0.7
Second lowest	56	23.9	178	76.1	
Middle	5	15.6	27	84.4	
High	3	20.0	12	80.0	

^a^Statistically significant

^b^Fisher’s exact test

**Table 4:** Multivariable logistic regression analysis for risk factors for anaemia in Gulu and Hoima Regional Hospitals

Variable	Number	Anaemic (%)	Crude OR (95 % CI)	AOR (95 % CI)
Age				
<20	199	47 (23.6)	1.0	1.0
20–24	243	48 (19.8)	0.8 (0.51–1.60)	1.8 (0.50–1.32)
25–29	178	42 (23.6)	1.0 (0.62–1.61)	1.1 (0.63–1.80)
30–34	86	21 (24.4)	1.0 (0.58–1.89)	1.3 (0.68–2.56)
35–39	31	4 (12.9)	0.5 (0.16–1.45)	0.6 (0.19–1.85)
≥40	6	2 (33.3)	1.6 (0.29–9.16)	1.4 (0.22–8.99)
Education				
No education	24	16 (32.7)	1.0	1.0
Primary	382	86 (22.1)	0.3 (0.15–0.81)	0.5 (0.27–1.10)
Secondary	283	55 (21.7)	0.3 (0.13–0.74)	0.6 (0.30–1.31)
Higher	547	7 (13.0)	0.2 (0.52–0.60)	0.4 (0.11–1.20)
Occupation				
Farmer	222	38 (17.1)	1.0	1.0
Trader	154	31 (20.1)	1.2 (0.72–2.07)	1.3 (0.73–2.21)
Formal employment	83	15 (18.1)	1.1 (0.55–2.07)	1.4 (0.63–2.96)
Handicraft	10	3 (30.0)	2.1 (0.51–8.44)	1.1 (0.25–4.45)
Housewife	274	77 (28.1)	1.9 (1.22–2.94)	1.7 (1.05–2.68)
Residence				
Rural	301	67 (22.3)	1.0	
Urban	442	97 (22.0)	1.0 (0.69–1.40)	
Marital status				
Single	79	14 (17.7)	1.0	1.0
Married	653	145 (22.2)	1.3 (0.72–2.43)	0.8 (0.43–1.64)
Widowed	4	2 (50)	4.6 (0.58–37.46)	2.0 (0.21–18.89)
Separated/divorced	7	3 (42.9)	3.0 (0.68–17.92)	3.0 (0.55–16.85)
District				
Hoima	387	47 (12.1)	1.0	1.0
Gulu	356	117 (32.9)	3.5 (2.40–5.23)	3.6 (2.41–5.58)
Trimester				
First	55	8 (14.6)	1.0	1.0
Second	305	63 (20.7)	1.5 (0.69–3.41)	1.5 (0.63–3.39)
Third	383	93 (24.3)	1.9 (0.86–4.14)	1.2 (0.52–2.74)
Gravidity				
1–4	610	133 (21.80)	1.0	
≥5	133	31 (23.3)	1.1 (0.70–1.70)	
Delivery gap				
None	250	61 (24.2)	1.0	
1–11	22	5 (22.7)	0.9 (0.32–2.58)	
12–24	116	23 (19.8)	0.8 (0.45–1.32)	
25–36	139	31 (22.3)	0.9 (0.54–1.46)	
>36	216	44 (20.4)	0.8 (0.51–1.23)	
Household size				
1–5	535	111 20.8	1.0	
6–10	190	48 25.3	1.3 (0.25–1.79)	
>10	18	5 27.8	1.5 (0.51–4.21)	
Wealth index				
Lowest	462	100 (21.7)	1.0	
Second lowest	234	56 (23.9)	1.1 (0.78–1.66)	
Middle	32	5 (15.6)	0.7 (0.25–1.79)	
High	15	3 (20.0)	0.9 (0.25–3.27)	
Still in school				
Yes	39	3 (7.7)	1.0	1.0
No	704	161 (22.9)	3.7 (1.11–12.10)	2.6 (0.73–9.26)

## Discussion

The overall prevalence of anaemia was 22.1 % which agrees with findings in Ethiopia and Nigeria, [16, 17] but lower than those reported elsewhere [18–21], and the WHO estimate of 40–60 % in developing countries [1]. The variations in the prevalence of anaemia may be due to the fact that the interventions employed to address anaemia in pregnancy vary in different settings.

The prevalence of anaemia was significantly higher in Gulu than in Hoima (*P* < 0.001). Gulu district was ravaged by a twenty-year war between the LRA and the Government of Uganda that left most of the social services in the district in ruins and a large majority of its population in poverty [22]. Our findings also show that being a housewife is an independent risk factor for anaemia. Because most housewives depend solely on their husbands’ earnings for their financial needs, the majority of them tend to be of low socio-economic status which has been reported as a known determinant of anaemia [23, 24]. This is also manifested by the finding that anaemia was more prevalent among women who had low monthly family income. Anaemia was also more prevalent among women who live in big households (>5 people) compared to those who live in small households. These might be low income families that were displaced from their family land during the LRA insurgency but have since decided to remain in the urban settings for a better life. Education level attained was also found to be associated with anaemia. In Uganda, low level of education is associated with unemployment, which consequently leads to poverty, a known risk factor for anaemia in pregnancy [21, 24].

Anaemia prevalence was highest (24.3 %) during the third trimester as compared to the first trimester (14.6 %) and second trimester (20.7 %). Haemodilution in pregnancy increases to peak during the second trimester which may explain the high prevalence of anaemia during this period. However, the increased incidence of anaemia during the third trimester may also indicate poor antenatal care and nutrition. These findings agree with that of Karaoglu and the WHO report [23, 25], but differ to those from Porto Novo, Cape Verde and Abeokuta, Nigeria [24, 26]. Although a study conducted in Trinidad and Tobago reported increased presence of anaemia with increasing gravidity [27], this study found no evidence of increased incidence of anaemia in a grand multigravid woman as compared to primigravid, secundigravid or multigravid woman. This finding is in agreement with other studies [16]. Perhaps the health education provided to pregnant women during antenatal visits leads to better health seeking behaviour and dietary habits, especially during pregnancy.

These findings will go a long way in addressing the problem of anaemia, which can affect psychological and physical behaviour. This is especially important because even very mild forms of anaemia have been reported to influence the sense of well being, lessen resistance to fatigue, lower productivity [28], aggravate other disorders, and affect work capacity [26]. In pregnant women, anaemia can result in increased risk of maternal and perinatal mortality, low birth weight, [29] and reduced resistance to blood loss with the result that death may occur from the blood loss associated with delivery. The strength of this study is the use of power formula to calculate the sample size and the random sampling of the study participants which enhances its generalisability.

### Limitations of this study

We did not consider other factors like parasitic infections which can lead to anaemia. We were therefore not able to determine their contribution to anaemia in our study population. Being a cross sectional study, we could not identify the cause and effect relationship.

## Conclusion

The prevalence of anaemia in pregnant women in Gulu is higher than in Hoima. Amongst pregnancy women, being a housewife is an independent risk factor for anaemia. Greater efforts are required to encourage early antenatal attendance from women in these at risk groups. This would allow iron and folic acid supplementation during pregnancy, which would potentially reduce the prevalence of anaemia.

## Additional file

Additional file 1: Questionnaire (English version). (PDF 1303 kb)

## Abbreviations

AOR: adjusted odds ratio
Hb: haemoglobin
HIV: human immuno-deficiency virus
LRA: Lord’s Resistance Army
WHO: World Health Organisation
