# Supplementary material for: Prevalence of anaemia and associated risk factors among pregnant women attending antenatal care in Gulu and Hoima Regional Hospitals in Uganda: A cross sectional study
Source: BMC Pregnancy Childbirth. 2016 Apr 11;16:76. doi: 10.1186/s12884-016-0865-4 (PMC4827189; doi:10.1186/s12884-016-0865-4)
Supplement: Additional file 1: — Questionnaire (English version). (PDF 1303 kb) [file 12884_2016_865_MOESM1_ESM.pdf]

Appendix 1: Questionnaire (English version)

We are conducting a study to determine the burden and reasons for anaemia among pregnant women attending antenatal care at Gulu and Hoima Regional Hospitals. The information obtained from this study will help health workers and the relevant authorities to find ways of reducing the problem of anaemia among pregnant women. We will therefore appreciate if you could answer the following questions very sincerely.

Thank you for accepting to take part in this study.

Date of interview: ....

Hospital name: ....

Participant's identification number: ....

A: Socio-demographic characteristics

1. How old are you? (age in completed years)
   i) 15 – 19 years
   ii) 20 – 24 years
   iii) 25 – 29 years
   iv) 30 – 34 years
   v) 35 – 39 years
   vi) > 39 years
2. What is your marital status?
   i. Single
   ii. Married
   iii. Widowed
   iv. Separated/divorced
3. Are you still attending school?
   i. Yes
   ii. No

GULU UNIVERSITY
INSTITUTIONAL REVIEW COMMITTEE
APPROVED
27 MAY 2012
FACULTY OF MEDICINE
P. O. Box 166, Gulu

4. What is the highest level of formal education you have attained?
    i) No formal education
    ii) Primary
    iii) Secondary
    iv) Tertiary
5. What is your occupation?
    i. Housewife
    ii. Farming
    iii. Trade
    iv. Handicraft
    v. Formal employment

B: Information on gravidity/parity

6. How old is this pregnancy?
    i) 1 – 12 weeks (first trimester)
    ii) 13 – 27 weeks (second trimester)
    iii) 28 - 40 weeks (trimester)
7. How many times have you been pregnant/gravid including this current pregnancy?
    i) 1 -4
    ii) ≥5
8. When did you last give birth?
    i) Never given birth
    ii) 1-11 months ago
    iii) 12 -24 months ago
    iv) 25 – 36 months ago
    v) >36 months ago
9. Is your home located in a rural or urban area? (*ask for village and parish name*)
    i) Urban
    ii) Rural

GULU UNIVERSITY
INSTITUTIONAL REVIEW COMMITTEE
APPROVED
★ 27 MAY 2012 ★
FACULTY OF MEDICINE
P. O. Box 166, Gulu

10. How many people live in your household?

i) 1 -5 people

ii) 6 – 10 people

iii) >10 people

C: Housing, sanitation, and family income

11. What type of building do you live in?

i. Mud and wattle

ii. Semi permanent building

iii. Permanent building

12. Who owns the building you live in?

i) Rented

ii) Owned by self

13. Does your household own a latrine/toilet?

i) Yes

ii) No

14. What is the total average monthly income (UGX) of your household?

i. <75,000

ii. 75,000 – <150,000

iii. 150,000 -300,000

iv. >300,000

Name of interviewer: ……………………………………………

Signature: …………………………………………...

GULU UNIVERSITY
INSTITUTIONAL REVIEW COMMITTEE
APPROVED
★ 27 MAY 2012 ★
FACULTY OF MEDICINE
P. O. Box 166, Gulu

Participant's ID number (as above): ....

Date: ....

**parameter**	**level**	**units**
Hb		g/dL
haematocrit		%
Red blood cell count		X106 /μL
MCV		fL
MCH		pg
MCHC		g/dL

Name of person entering data: ....

Signature: ....

GULU UNIVERSITY
INSTITUTIONAL REVIEW COMMITTEE
APPROVED
★ 27 MAY 2012 ★
FACULTY OF MEDICINE
P. O. Box 166, Gulu
